# Meta-Analysis of the Association between Vitamin D and Autoimmune Thyroid Disease

**DOI:** 10.3390/nu7042485

**Published:** 2015-04-03

**Authors:** Jiying Wang, Shishi Lv, Guo Chen, Chenlin Gao, Jianhua He, Haihua Zhong, Yong Xu

**Affiliations:** Department of Endocrinology and Metabolism, Affiliated Hospital of Luzhou Medical College, Luzhou 646000, China; E-Mails: wangjiying90@aliyun.com (J.W.); lss219219@163.com (S.L.); terry1210@aliyun.com (G.C.); gaochenlin00@126.com (C.G.); hyjx88@163.com (J.H.); zhh-302102@163.com (H.Z.)

**Keywords:** vitamin D, autoimmune thyroid disease, Graves’ disease, Hashimoto thyroiditis, meta-analysis

## Abstract

Although emerging evidence suggests that low levels of vitamin D may contribute to the development of autoimmune disease, the relationship between vitamin D reduction and autoimmune thyroid disease (AITD), which includes Graves’ disease (GD) and Hashimoto thyroiditis (HT), is still controversial. The aim was to evaluate the association between vitamin D levels and AITD through systematic literature review. We identified all studies that assessed the association between vitamin D and AITD from PubMed, Embase, CENTRAL, and China National Knowledge Infrastructure (CNKI) databases. We included studies that compared vitamin D levels between AITD cases and controls as well as those that measured the odds of vitamin D deficiency by AITD status. We combined the standardized mean differences (SMD) or the odds ratios (OR) in a random effects model. Twenty case-control studies provided data for a quantitative meta-analysis. Compared to controls, AITD patients had lower levels of 25(OH)D (SMD: −0.99, 95% CI: −1.31, −0.66) and were more likely to be deficient in 25(OH)D (OR 2.99, 95% CI: 1.88, 4.74). Furthermore, subgroup analyses result showed that GD and HT patients also had lower 25(OH)D levels and were more likely to have a 25(OH)D deficiency, suggesting that low levels of serum 25(OH)D was related to AITD.

## 1. Introduction

Because an estimated one billion people worldwide have vitamin D deficiency or insufficiency [1], vitamin D has become an important focus of current medical research. Although the biological activities of vitamin D are mainly manifested in the regulation of calcium-phosphorus metabolism, studies in the past 30 years indicate vitamin D may play an important role in the immune system [2,3]. Results show that 1,25-dihydroxyvitamin D3 can either prevent or markedly suppress experimental autoimmune encephalomyelitis, rheumatoid arthritis, systemic lupus erythematosus, type 1 diabetes, and inflammatory bowel disease [4,5,6,7,8]. Clinical trials have also shown that vitamin D supplements may reduce the incidence of rheumatoid arthritis, multiple sclerosis, and type 1 diabetes in children [1]. In the past two decades, vitamin D receptors have been found not only in bone, kidney, and intestine, but also in the immune system (T and B cells, macrophages, and monocytes), reproductive system, endocrine system, muscles, brain, skin, and liver [9], suggesting that the role of vitamin D is not limited to the skeletal system.

Recently, many studies have shown that low levels of vitamin D contribute to Graves’ disease (GD) and Hashimoto thyroiditis (HT) and that combining vitamin D with anti-thyroid drugs or thyroid hormone contributes to the treatment of autoimmune thyroid disease (AITD) by suppressing the autoimmune reaction and reducing serum levels of thyroid autoantibodies [10,11]. However, other authors have proposed that vitamin D deficiency does not increase the risk of AITD and is not associated with early-stage AITD [12,13]. Because the association between vitamin D levels and AITD is still controversial, we conducted a systematic review of the published studies that investigated the relationship between serum 25(OH)D levels and AITD.

## 2. Methods

### 2.1. Bibliographic Search

A bibliographic search was performed on PubMed, Embase, CENTRAL, and China National Knowledge Infrastructure (CNKI) (updated to 20 December 2014) by two investigators (Jiying Wang and Shishi Lv) using the key words “vitamin D”, in combination with “autoimmune thyroid disease”, “thyroid autoimmunity”, “Graves’ disease” or “Hashimoto thyroiditis”. Articles were only considered if they were in English or Chinese and were not hand-searched.

### 2.2. Eligibility Criteria and Excluded Studies

Articles were included in this meta-analysis if (1) they described a population-based case-control study; (2) the case group consisted of AITD patients and the control group included healthy individuals; (3) the outcome measures reported quantitative vitamin D levels (mean ± SD) and qualitative vitamin D levels (odds of vitamin D deficiency); (4) the study was a high-quality study (≥7 points according to the Cochrane’s Newcastle-Ottawa Scale evaluation standard for case-control studies [14]); and (5) was written in English or Chinese. After reading the title and abstract, we excluded a study if it was an animal or *in vitro* experiment, did not contain original data (e.g., was a medical recapitulate), was not related to AITD, did not contain data on vitamin D, or was not a case-control study, case reports, and studies consisting of duplicate data. After reading the full text, we excluded from the study if the comparator group did not conform to the requirements (e.g., compared female patients and male patients), duplicate publication, conference abstracts, no data about vitamin D level (mean ± SD), inconsistent data, or it did not refer to AITD. Disagreement was resolved by discussion between the authors (Jiying Wang and Shishi Lv). If they could not reach a consensus, another investigator (Yong Xu) was consulted regarding the disagreements.

### 2.3. Data Extraction

The following information was extracted from each study: the author, publication year, participant characteristics (age, gender, number), season, type of serum vitamin D assay, serum vitamin D (reported in ng/mL; for studies that reported vitamin D in nmol/L, we converted the values to ng/mL by dividing by 2.496 [15]), the number of patients with vitamin D deficiency, the cut-off for defining vitamin D deficiency, *p* value, and study quality. Information was independently extracted by Jiying Wang and Guo Chen, and all data were confirmed by another author (Chenlin Gao).

### 2.4. Statistical Method

For studies that reported quantitative vitamin D levels for AITD participants and controls, we combined the standardized mean differences (SMD) in a random effects model. For studies that reported qualitative vitamin D levels, we pooled the odds ratios (OR) in a random effects model. We assessed statistical heterogeneity using *Q*-tests and the I^2^ statistic. Publication bias was assessed using Egger’s test (*p* < 0.1 was considered to be publication bias). All analyses were carried out using the commands metan and metabias in Stata software, version 12.0 (Stata Corp).

## 3. Results

**Figure 1:**
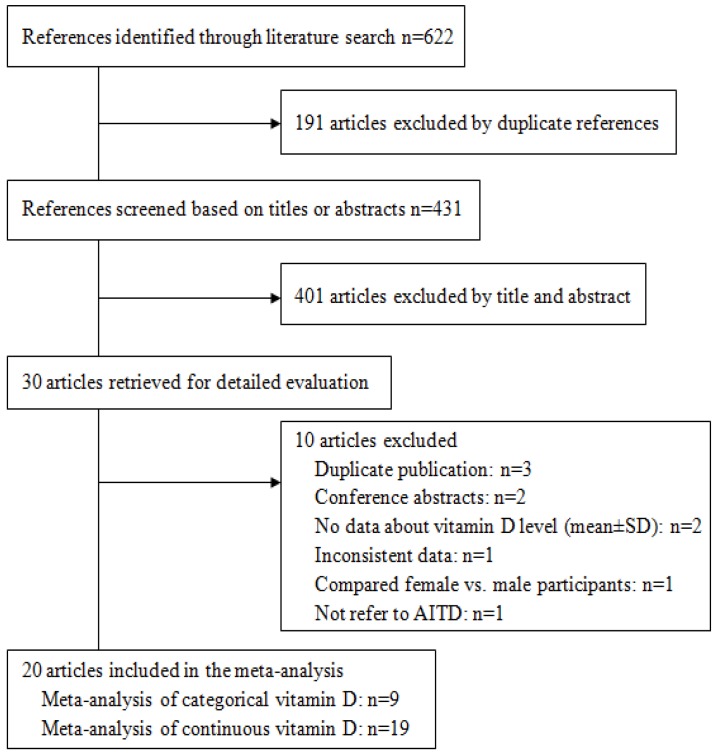
Flow diagram showing study selection.

Our search identified 431 unique references, of which 411 did not meet our inclusion criteria. We conducted meta-analyses on the remaining 20 articles [10,11,13,16,17,18,19,20,21,22,23,24,25,26,27,28,29,30,31,32]. (Figure 1). Of the 20 included articles, 19 were used to analyze continuous data on vitamin D levels (Table 1) and nine were used to analyze dichotomous data on vitamin D (deficiency or no deficiency) (Table 2).

Overall, most studies showed a higher prevalence of vitamin D deficiency and lower vitamin D levels in AITD patients compared with controls.

**Figure 2:**
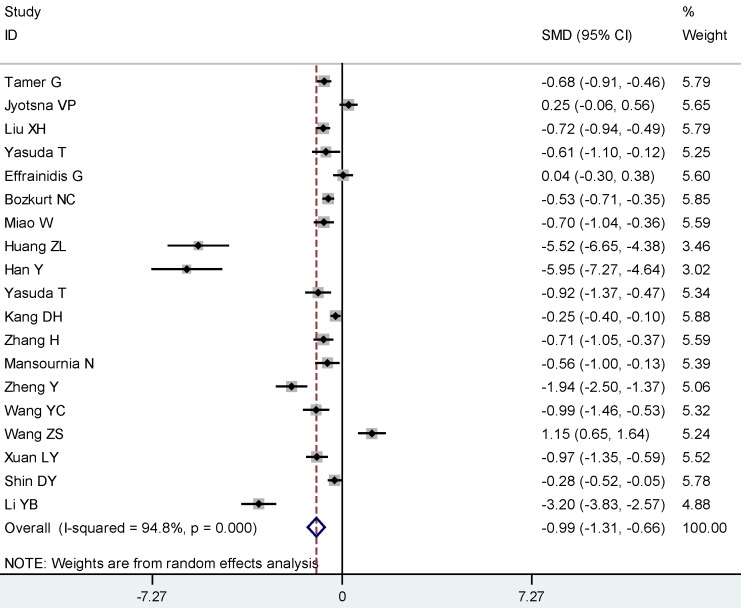
Meta-analysis of studies (chronologically ordered) reporting 25(OH)D levels in autoimmune thyroid disease (AITD) *vs.* controls, standardized mean difference with 95% confidence interval.

The meta-analysis of the continuous vitamin D by AITD status included 3603 participants (1782 AITD cases and 1821 controls). On average, AITD patients had lower levels of 25(OH)D compared to controls (SMD: −0.99, 95% CI: −1.31, −0.66) (I^2^ 94.8%, *p* < 0.01) (Figure 2). We found evidence of publication bias as evidenced by Egger’s test (*p* = 0.009).

**Figure 3:**
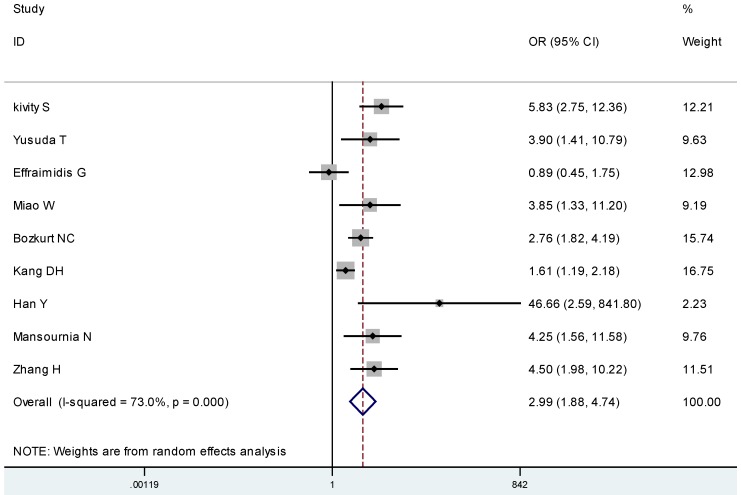
Meta-analysis of studies (chronologically ordered) reporting dichotomous data on 25(OH)D levels in autoimmune thyroid disease (AITD) *vs.* controls and estimated odds ratios (ORs) with 95% confidence interval.

For the presence of vitamin D deficiency, nine studies totaling 994 AITD participants and 1035 controls were included. AITD participants were more likely to be deficient in 25(OH)D (OR 2.99, 95% CI: 1.88, 4.74) (I^2^ 73.0%, *p* < 0.01) compared to their controls (Figure 3). We found evidence of publication bias as evidenced by Egger’s test (*p* = 0.056).

**Figure 4:**
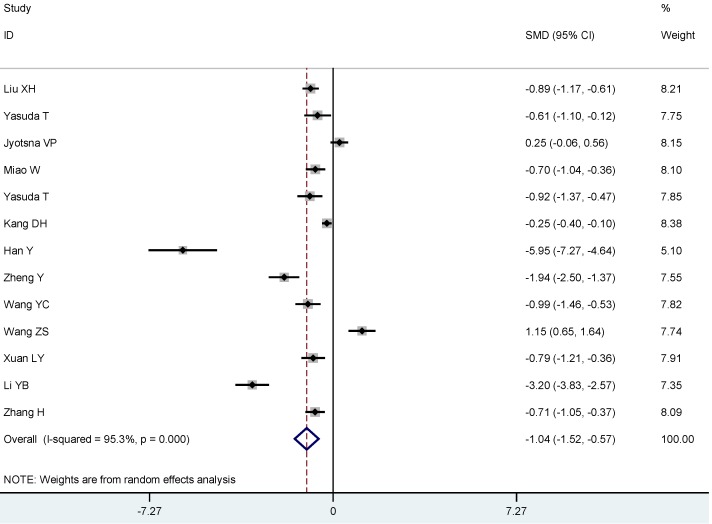
Meta-analysis of studies (chronologically ordered) reporting 25(OH)D levels in Graves’s disease *vs.* controls, standardized mean difference with 95% confidence interval.

**Figure 5:**
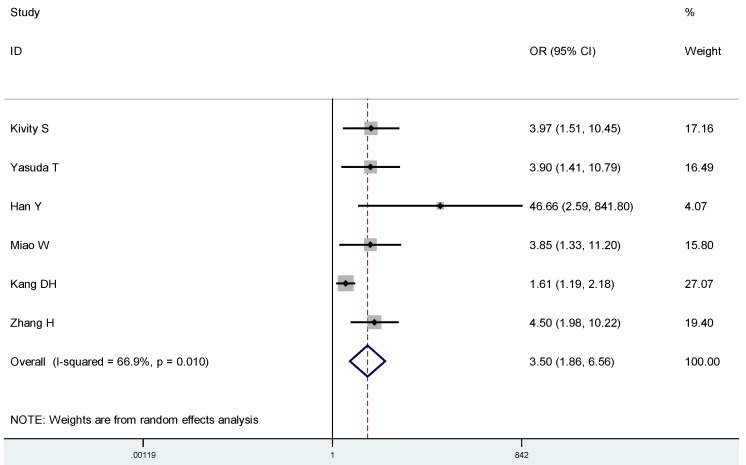
Meta-analysis of studies (chronologically ordered) reporting dichotomous outcomes of 25(OH)D levels in Graves’ disease *vs.* controls and estimated ORs with 95% confidence interval.

**Figure 6:**
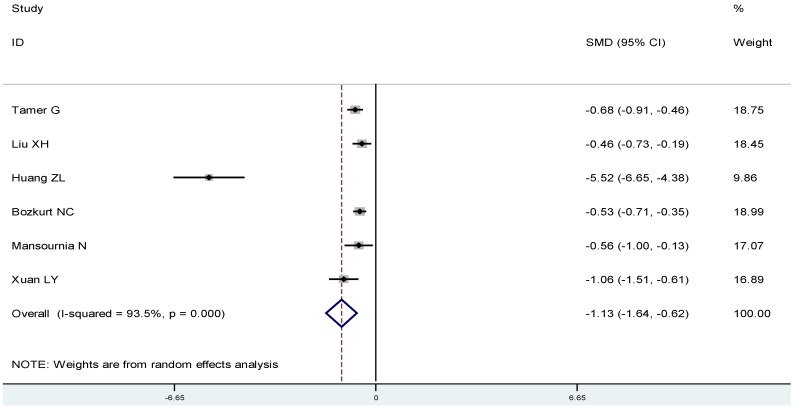
Meta-analysis of studies (chronologically ordered) reporting 25(OH)D levels in Hashimoto thyroiditis *vs.* controls, standardized mean difference with 95% confidence interval.

**Figure 7:**
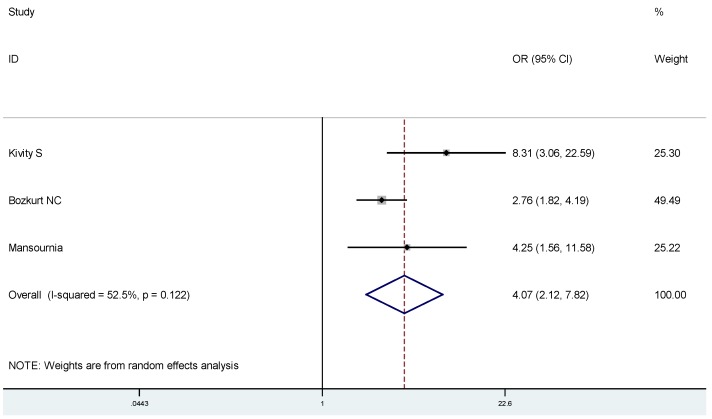
Meta-analysis of studies (chronologically ordered) reporting dichotomous data of 25(OH)D levels in Hashimoto thyroiditis *vs.* controls and estimated ORs with 95% confidence interval.

To estimate the association between 25(OH)D and Graves’ disease or Hashimoto thyroiditis, respectively, we conducted subgroup analyses: On average, Graves’ disease patients had lower 25(OH)D compared to controls (SMD: −1.04, 95% CI: −1.52, −0.57) (Figure 4), and were more likely to have a 25(OH)D deficiency(OR 3.50, 95% CI: 1.86, 6.56) (Figure 5). Likewise, Hashimoto thyroiditis patients had lower 25(OH)D compared to controls (SMD: −1.13, 95% CI: −1.64, −0.62) (Figure 6), and were more likely to have a 25(OH)D deficiency (OR 4.07, 95% CI: 2.12, 7.82) (Figure 7).

**Table 1:** Studies with continuous data on vitamin D levels in AITD and controls.

First Author and Year	AITD (*N*)/Total (*N*)	AITD, males, %	AITD, Year, mean ± SD	Assay Method	Season of Collected Samples	25(OH)D in AITD, ng/mL mean ± SD	25(OH)D in Control, ng/mL mean ± SD	*p*-Value	Quality of Study (Score)
Yusuda T 2013	36/85	0	37.8 ± 8.1	CPBA	Sum, A	14.5 ± 2.9	18.6 ± 5.3	<0.0005	8
Tamer G 2011	161/323	6/161	35.4 ± 7.9	RIA	W	16.3 ± 10.4	29.6 ± 25.5	<0.0001	9
Yusuda T 2012	26/72	0	37.3 ± 13.0	CPBA	W, S	14.4 ± 4.9	17.1 ± 4.1	<0.05	8
Bozkurt NC 2013	360/540	114/360	42.55 ± 11.35	ELISA	Sum	12.2 ± 5.6	15.4 ± 6.8	<0.001	8
Effraimidis G 2012	67/134	NG	38.3 ± 11.5	RIA	ALL	21.6 ± 9.2	21.2 ± 9.3	NS	8
Han Y 2013	30/50	6/30	35.7 ± 7.3	HPLC	W, S	17.51 ± 6.14	58.84 ± 8.01	<0.01	7
Miao W 2013	70/140	22/70	40 ± 15.2	ECLIA	W, S	12.7 ± 5.25	16.56 ± 5.8	<0.01	9
Huang ZL 2013	40/60	6/40	44.6 ± 8.5	ECLIA	S, A	16.26 ± 4.16	49.5 ± 8.68	<0.01	8
Liu XH 2012	160/325	25/160	43.25 ± 8.55	ECLIA	W, S, Sum	13.51 ± 5.88	19.48 ± 10.12	<0.05	8
Xuan LY 2014	89/134	32/89	33.92 ± 12.70	ELISA	ALL	19.04 ± 9.72	29.95 ± 13.86	<0.01	7
Shin DY 2014	111/304	21/111	48.7 ± 12.7	RIA	ALL	12.6 ± 5.5	14.5 ± 7.3	<0.001	8
Li YB 2014	40/90	0	34 ± 14	ELISA	W, S	13 ± 5	29 ± 5	<0.05	8
Zhang H 2014	70/140	28/70	31.77 ± 10.32	ELISA	S	21.15 ± 4.41	24.28 ± 4.37	<0.05	8
Jyotsna VP 2012	80/160	18/80	36.33 ± 11.15	RIA	ALL	12.67 ± 6.24	10.99 ± 7.05	<0.05	7
Mansournia N 2014	41/86	NG	42.3 ± 15.3	HPLC	A	15.9 ± 12.1	24.4 ± 17.3	<0.01	8
Zheng Y 2014	33/72	14/33	35.3 ± 9.23	ELISA	ALL	15.71 ± 6.79	30.84 ± 8.57	<0.01	7
Wang YC 2014	60/90	22/60	35.1 ± 7.95	ECLIA	W, S, Sum	12.28 ± 5.83	18.1 ± 5.92	<0.01	7
Kang DH 2013	280/719	100/280	42.5 ± 7.9	ELISA	A	21.68 ± 9.54	24.05 ± 9.58	<0.01	7
Wang ZS 2014	28/79	0	NG	ECLIA	ALL	26.98 ± 9.02	19.05 ± 5.47	<0.01	7

(Introductions of Table 1: (1) Assay method: ELISA, enzyme-linked immunosorbent assay; HPLC, high performance liquid chromatography; ECLIA, chemiluminescence immunoassay; CPBA, competitive protein binding assay; RIA, radioimmunoassay; (2) Season: S, spring; Sum, summer; A, autumn; W, winter. (3) *N*, number; NG, not given; NS, not significant.)

**Table 2:** Studies with dichotomous data on vitamin D deficiency and no deficiency in AITD and controls.

First Author and Year	AITD(*N*)/Total (*N*)	AITD, Males, %	AITD, year (Mean or Range)	Assay Method	Season of Collected Samples	25(OH)D Deficiency in AITD (N)	25(OH)D Deficiency in Control (N)	Criterion of 25(OH)D Deficiency	*p*-Value	Quality of Study (Score)
Yusuda T 2012	26/72	0	37.3 ± 13.0	CPBA	W, S	17	15	<15 ng/mL	<0.05	8
Bozkurt NC 2013	360/540	57/180	42.55 ± 11.35	ELISA	Sum	150	37	<10 ng/mL	<0.001	8
Effraimidis G 2012	67/134	NG	38.3 ± 11.5	RIA	ALL	33	23	<20 ng/mL	=0.05	8
HanY 2013	30/50	6/30	35.7 ± 7.3	HPLC	W, S	16	0	<20 ng/mL	<0.01	7
Miao W 2013	70/140	22/70	40 ± 15.2	ECLIA	W, S	65	54	<20 ng/mL	<0.05	9
Kivity S 2011	50/148	6/50	45 ± 16	DCCLIA	S	35	37	<10 ng/mL	<0.001	8
Zhang H 2014	70/140	28/70	31.77 ± 10.32	ELISA	S	30	10	<20 ng/mL	<0.05	8
Kang DH 2013	280/719	100/280	42.5 ± 7.9	ELISA	A	133	158	<20 ng/mL	<0.01	7
Mansourria N 2014	41/86	NG	42.3 ± 15.3	HPLC	A	34	24	<20 ng/mL	0.82	8

(Introductions of Table 2: (1) Assay method: ELISA, enzyme-linked immunosorbent assay; HPLC, high performance liquid chromatography; ECLIA, chemiluminescence immunoassay; CPBA, competitive protein binding assay; DCCLIA, direct competitive chemiluminescence immunoassay; RIA, radioimmunoassay. (2) Season: S, spring; Sum, summer; A, autumn; W, winter. (3) *N*, number; NG, not given; NS, not significant.)

## 4. Discussion

The association between low serum vitamin D and autoimmune diseases has been generally accepted by researchers. Bellastella G. found that automimmune disease patients showed 25(OH)D levels significantly lower than healthy controls [33]. A meta-analysis of vitamin D receptor gene polymorphisms and AITD showed a significant correlation between certain vitamin D receptor gene polymorphisms (such as *Bsm*I and *Taq*I) and autoimmune thyroid diseases [34], but no meta-analysis of serum vitamin D levels and AITD has been published to date. In the present study, the serum 25(OH)D was lower in AITD patients compared to healthy control individuals, and AITD was more likely to develop in individuals who showed serum 25(OH)D deficiencies, which suggested that vitamin D deficiency may play a role in the pathological process of AITD.

AITD has been traditionally thought to be related to unbalanced ratio of T helper cell type 1 (Th1) and Th2 cells. Graves’ disease occurs when a high proportion of Th2 cells are present and secrete the cytokine IL-4 [35,36,37], and a complete lack of IL-4 has been shown to eliminate Graves’ disease in animal model [38]. Conversely, Hashimoto thyroiditis patients have a high proportion of Th1 cells, which secrete the cytokine IFN-γ [39]. Recent studies showed the secretion of cytokines from Th17 is involved in the development of AITD [40,41]. IF-γ and IF-17A mRNA expression is significantly higher in Hashimoto thyroiditis patients than in healthy controls [42,43]. Interestingly, vitamin D plays an important role in regulating Th1, Th2, and Th17 cells, as well as the secretion of IFN-γ, IL-4, and IL-17 [44,45,46,47]. These findings may explain why lower levels of vitamin D contribute to thyroid gland immune disorder. On the other hand, Graves’ disease is an autoimmune thyroid disorder in which thyrotrophin receptor antibody (TRAb) causes hyperthyroidism [48]. Low vitamin D status is associated with increased TRAb in this disease [22]. Results also show that levels of 25O (HD) <50 nmol/L are a risk factor for positive thyroid autoantibody (Thyroid peroxidase antibody (TPOAb) and thyroglobulin antibodies (TgAb)) [49]. Thus, this increased thyroid autoantibody in AITD, may be a consequence of the lower levels of vitamin D contributes to AITD.

The levels of vitamin D may dictate the prognosis of Graves’ disease [50], and may create an opportunity for vitamin D supplementation for patients? Research by Kawakami-Tani shows that concomitant administration (such as thyroid hormones or anti-thyroid drugs) of 1α(OH)D3 is useful for treating hyperthyroidism in patients with Graves’ disease [51]. Moreover, preliminary results of a small randomized controlled trial also showed that vitamin D treatment significantly decreased TPOAb and TgAb compared with placebo treatment in AITD patients [52]. Current evidence, however, is not definitive, the cost-effectiveness of vitamin D supplementation in AITD patients, as well as its optimal safe doses require further investigation.

To our knowledge, this was the first meta-analysis to investigate the association between vitamin D levels and AITD. The inclusion of Embase, PubMed, CENTRAL, and the CNKI database added strength to our study. However, our study had some limitations. Firstly, many of the original studies did not adjust for potentially important confounders, such as season or assay method. The prevalence of vitamin D deficiency and mean 25(OH)D levels did not distinctly different between winter and summer weather [53]. Limitations in reaching significant difference may be due to interassay and interlaboratory variability in measurements of vitamin D [54], the cut-off for defining vitamin D deficiency and the method of AITD diagnosis, which varied across studies, along with the language differences among the studies and publication bias may have contributed to the heterogeneity of our findings. Criterion of vitamin D deficiency include <10 ng/mL, <15 ng/mL and <20 ng/mL, but result showed that cut-points of vitamin D deficiency should be assay specific rather than universal and that greater consistency between laboratories is required [53]. In the studies, AITD was diagnosed by thyroid function test, anti-thyroid antibodies, with or without ultrasonography. Thirdly, due to the nature of the abstracted case control studies in our review, further prospective studies are needed to clarify whether reduced vitamin D level is a causal factor in the pathogenesis of autoimmune diseases or a consequence of this. Finally, we found statistical heterogeneity in our analysis. However, we did not find any major clinical heterogeneity and therefore the pooled analysis was appropriate for our study.

## 5. Conclusions

In conclusion, we have demonstrated that vitamin D deficiency is prevalent in AITD subjects and that these subjects have lower levels of serum 25(OH)D, suggesting that lower serum vitamin D is related to AITD and the deficiency in vitamin D may plays a role in the development of the disease. Large-sample multi-center randomized controlled trials will help to consolidate whether there is an association between vitamin D and AITD, and consequently give directions as to the beneficial effect of vitamin D supplementation in those patients.
